# Circular RNA 100146 Promotes Colorectal Cancer Progression by the MicroRNA 149/HMGA2 Axis

**DOI:** 10.1128/MCB.00445-20

**Published:** 2021-01-25

**Authors:** Kunpeng Liu, Yuhua Mou, Xiufang Shi, Tingkun Liu, Zhanfeng Chen, Xingmei Zuo

**Affiliations:** a Department of General Surgery, Rizhao People's Hospital, Rizhao City, China; b Department of Pediatrics, Chinese Medicine Hospital, Rizhao City, China; c Rizhao City Tuberculosis Control Center, Rizhao City, China; d Department of General Surgery, Rizhao People's Hospital, Rizhao City, China

**Keywords:** colorectal cancer, circRNA 100146, miR-149, HMGA2

## Abstract

Colorectal cancer (CRC) has developed into the third leading cause of cancer-associated death worldwide. Studies have confirmed that circular RNAs (circRNAs) absorb microRNAs (miRNAs) to regulate the function of downstream genes. This study aimed to explore the underlying mechanism of circRNA 100146 in CRC. The expression of circRNA 100146, miRNA 149 (miR-149), and high mobility group AT-Hook 2 (HMGA2) was detected by quantitative real-time PCR (RT-qPCR). A series of biofunctional effects (cell viability, apoptosis, migration/invasion) were evaluated by the use of methyl thiazolyl tetrazolium (MTT), flow cytometry, and transwell assays. Protein levels were measured by Western blot assay. A xenograft model was established for *in vivo* experiments. The interactions among circRNA 100146, miR-149, and HMGA2 were evaluated by dual-luciferase reporter assay, RNA immunoprecipitation assays, or RNA pulldown assay. circRNA 100146 was upregulated in CRC tissues and cells. circRNA 100146 knockdown inhibited cell proliferation, promoted apoptosis, and suppressed migration and invasion *in vitro* and impeded tumor growth *in vivo*. Also, miR-149 was negatively regulated by circRNA 100146 and was targeted to HMGA2 and mediated its expression. Moreover, miR-149 interference abrogated the activities of silenced circRNA 100146 in proliferation, apoptosis, migration, and invasion. Furthermore, HMGA2 overexpression abated the effects described above caused by circRNA 100146 silencing, while the mutations on miR-149 binding sites in the 3′ untranslated region (3′-UTR) of HMGA2 led to its loss of this ability. circRNA 100146 knockdown repressed proliferation, enhanced apoptosis, and hindered migration and invasion in SW620 and SW480 cells through targeting the miR-149/HMGA2 axis.

## Abstract (precis)

Colorectal cancer (CRC) has developed into the third leading cause of cancer-associated death worldwide. Studies have confirmed that circular RNAs (circRNAs) absorb microRNAs (miRNAs) to regulate the function of downstream genes.

## INTRODUCTION

Colorectal cancer (CRC) ranks third among all causes leading to cancer-related death globally, with an incidence of more than 1 million cases and mortality of about 600,000 patients annually (1, 2). In general, patients have reached the late stage (III or IV) of CRC by the time that they are diagnosed with the disease, and most patients do not have enough time to receive standard treatment and eventually succumb to the disease (3). The other reason for the high mortality rate of CRC patients is the lack of effective therapeutic approaches for the treatment of CRC. At present, the common therapeutic methods include radiotherapy, chemotherapy, and surgical resection, while the mortality of CRC patients remains unsatisfactory high, even among patients who had received treatment in time (4). Therefore, an early diagnostic biomarker and a feasible therapeutic target are urgently needed for improving the survival rate of CRC patients.

Circular RNAs (circRNAs) are a new kind of noncoding RNAs, and some of them actually have the potential to code peptides or proteins (5). It has been reported that circRNAs were found to regulate gene expression and repress tumorigenesis of multiple cancers. Unlike message RNA, they have three-dimensional covalently closed loops instead of polyadenylated tails and polarities (6). Increasingly, studies have indicated that circRNAs were identified as regulators in RNA splicing and transcription and also played vital roles in physiological processes of human cancers (7, 8). circRNA 100146, derived from exon 5 and exon 6 of the EIF3I gene, which is also identified as hsa_circ_0011385 in the circBase, was shown to have an oncogenic role in non-small-cell lung cancer, and it absorbed microRNA 361-3p (miR-361-3p) and miR-615-5p to promote cell progression (9). Moreover, high-throughput microarray studies have disclosed that hsa_circ_0011385 was highly expressed in CRC tissues in contrast with the normal (healthy) tissues based on the data from Gene Expression Omnibus databases (GEO, NIH; accession no. GSE126094 and GPL19978). However, the effect and underlying mechanism of circRNA 100146 in CRC were still unknown.

MicroRNAs (miRNAs) also belong to noncoding RNAs with the length of 18 to 24 nucleotides (nt). miRNAs can bind to the 3′ untranslated region (3′-UTR) of target mRNAs and suppress the expression of mRNA, resulting in the loss of many biological functions. It has been widely reported that miRNAs have been found to be involved in many diseases such as nervous disease, kidney disease, heart disease, and cancer (10). Emerging studies revealed that miR-149 functioned as a suppressor in CRC and repressed the migration and invasion of CRC cells by targeting FOXM1 (11, 12). Although the role of miR-149 has been explored, the mechanism of circRNA 100146 regulating miR-149 remains to be elucidated.

High mobility group AT-Hook 2 (HMGA2) is a member of the high-mobility group (HMG) family and is located in the region of chromosome 12 (13). Protein encoded by HMGA2 gene has no transcription function, while HMGA2 can modulate downstream gene expression through binding to AT-rich minor grooves via AT-hook DNA binding domains (14, 15). HMGA2 was reported to participate in cell progression in plenty of cancers, including gastric cancer, breast cancer, lung cancer, and CRC (16–19). Despite much research performed to explain the role of HMGA2 in CRC, how HMGA2 participates in the regulation of CRC needs to be further studied.

In the current study, we detected the expression and underlying mechanism of circRNA 100146 in CRC. circRNA 100146 was highly expressed in CRC patients and cells. circRNA 100146 silencing hindered cell progression in CRC *in vitro* and *in vivo*. We also confirmed that circRNA 100146 targets to miR-149 to regulate the expression of HMGA2, which eventually affected the progression of CRC cells. This research provided theoretical support for the idea of circRNA 100146 serving as an early diagnostic biomarker for detection of CRC.

## RESULTS

### circRNA 100146 was overexpressed in CRC patients and cells, and its levels were decreased by si-circRNA 100146 in CRC cells.

**FIG 1:**
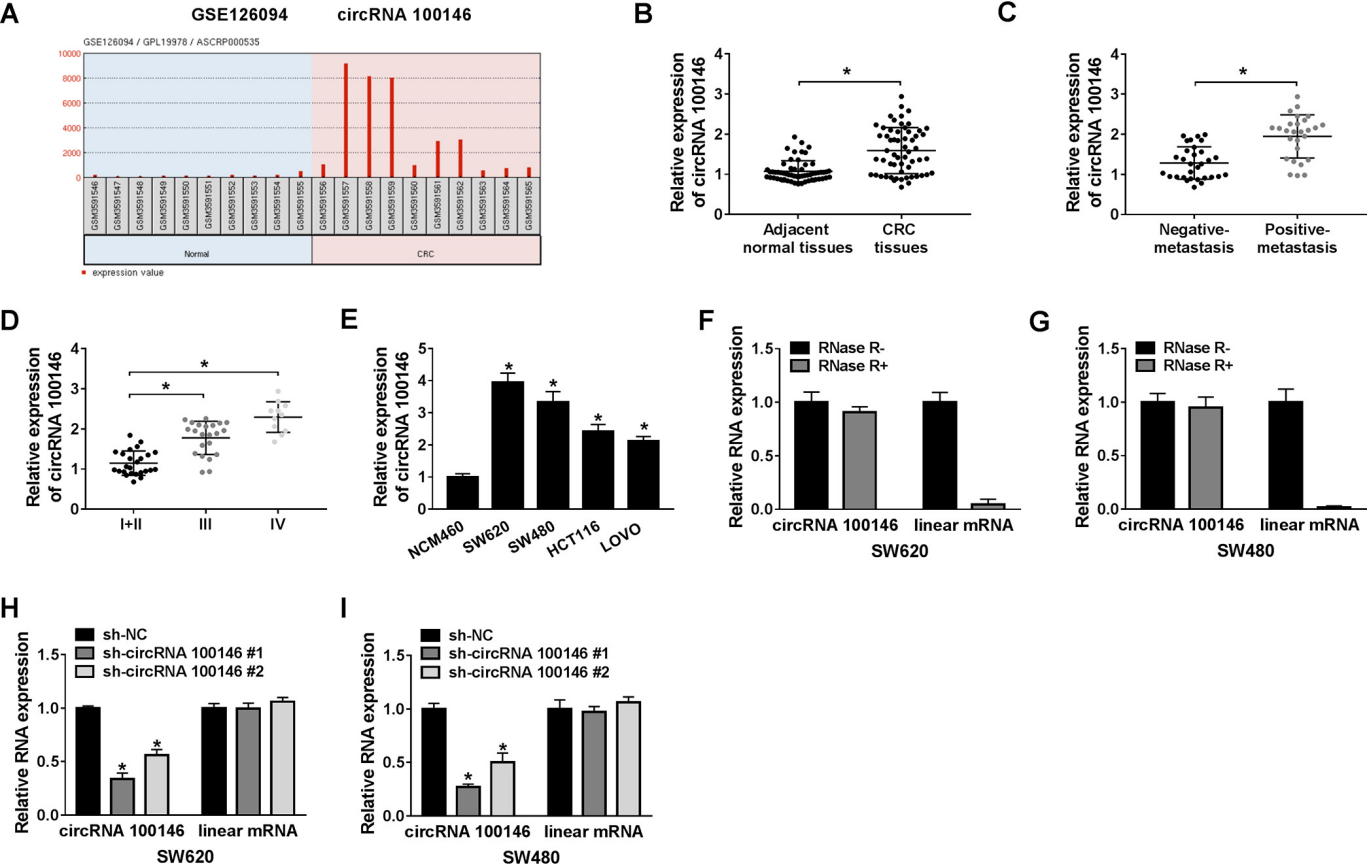
circRNA 100146 was upregulated in CRC tissues and cells. (A) Expression of circRNA 100146 in normal subjects and CRC tissues based on the data from GEO databases (accession no. GSE126094 and GPL19978). (B) The expression of circRNA 100146 was detected by RT-qPCR assay in CRC tissues and adjacent normal tissues. (C) circRNA 100146 expression was measured by RT-qPCR assay in positive and negative metastasis patients. (D) circRNA 100146 expression was detected by RT-qPCR assay in patients at stage I + II, III, and IV. (E) The expression of circRNA 100146 was measured by RT-qPCR assay in SW620, SW480, HCT116, and LOVO cells and normal colonic epithelial cell line NCM460. (F and G) RT-qPCR assay was conducted after RNA digested with or without RNase R in SW620 (F) and SW480 (G) cells. (H and I) RT-qPCR assay was performed in SW620 (H) and SW480 (I) cells transfected with sh-NC, sh-circRNA 100146 no. 1, or sh-circRNA 100146 no. 2. *, *P < *0.05.

On the basis of data from GEO databases (accession no. GSE126094 and GPL19978), we found that circRNA 100146 expression was enhanced in CRC tissues in contrast with normal tissues (Fig. 1A). Subsequently, the levels of expression of circRNA 100146 in 58 CRC patients were examined. Quantitative real-time PCR (RT-qPCR) assay was conducted to detect the expression of circRNA 100146 in CRC patients (*n* = 58), and the results showed that circRNA 100146 expression was greatly increased in CRC tissues compared with adjacent normal tissues (Fig. 1B). circRNA 100146 expression in patients with positive metastasis was enhanced relative to that in patients with negative metastasis (Fig. 1C). Further, patients with disease in stages III and IV had higher circRNA 100146 expression than patients with disease in stages I and II and the highest level of expression was seen in stage IV patients (Fig. 1D). Also, the levels of expression of circRNA 100146 were elevated in SW620, SW480, HCT116, and LOVO cells compared with the normal colon cell line NCM460 (Fig. 1E). RNase R, a highly conserved 3′-to-5′ exoribonuclease, can digest almost all linear RNAs whereas circular RNAs are naturally resistant to RNase R due to its covalently closed structure (5). After digestion with RNase R, the level of expression of linear mRNA was significantly reduced, while circRNA 100146 expression remained unchanged in SW620 and SW480 cells (Fig. 1F and G). In addition, circRNA expression was greatly decreased by the activity of small interfering circRNA (si-circRNA) 100146 no. 1 or si-circRNA 100146 no. 2 in SW620 and SW480 cells, while they had no effect on the level of expression of linear mRNA (Fig. 1H and I). These results indicated that circRNA 100146 was upregulated in CRC patients and that short hairpin circRNA (sh-circRNA) 100146 sharply reduced circRNA 100146 expression.

### Knockdown of circRNA 100146 suppressed CRC progression *in vitro*.

**FIG 2:**
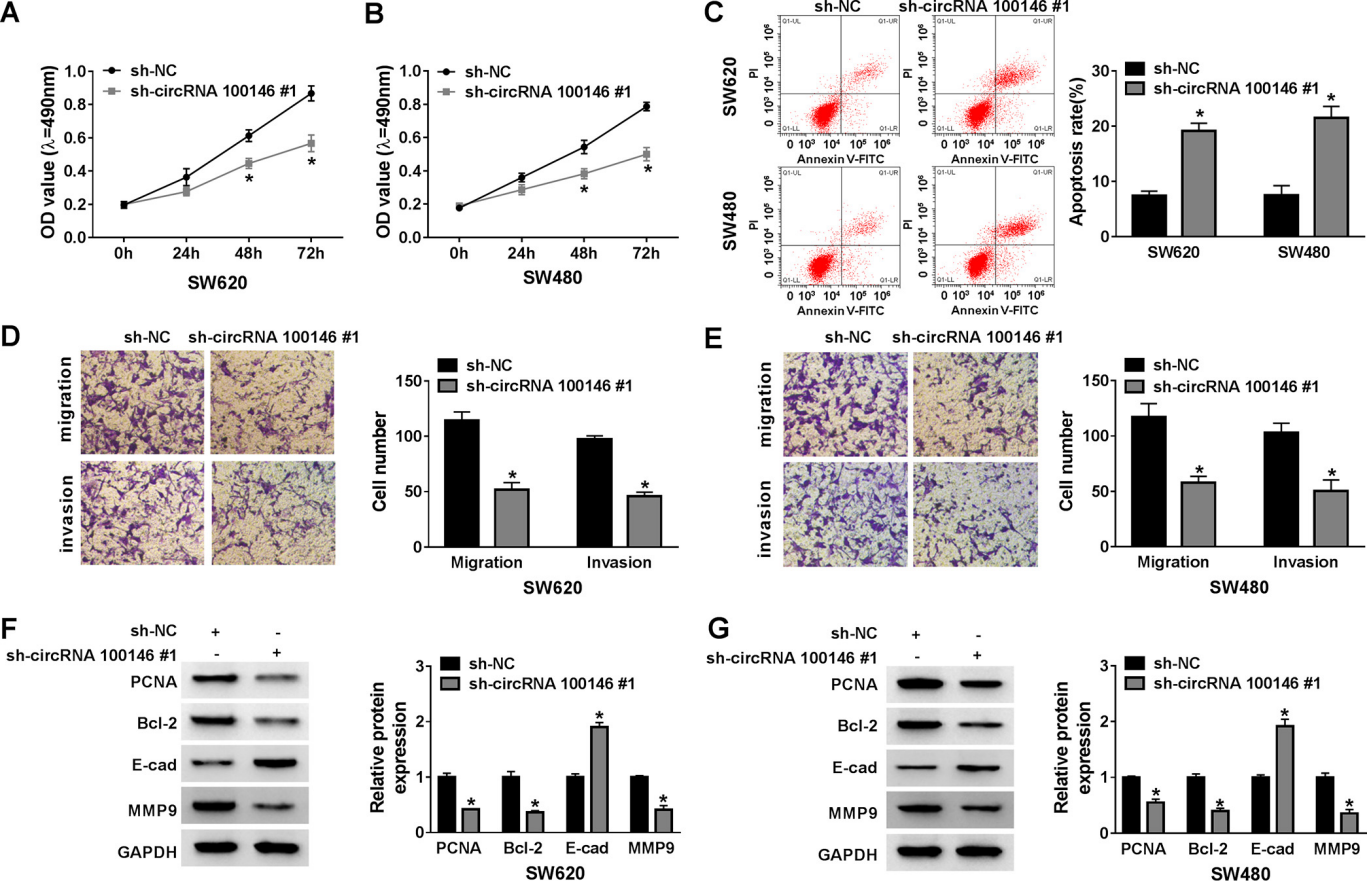
circRNA 100146 knockdown hindered CRC cell growth *in vitro*. (A and B) The cell viability of SW620 and SW480 cells transfected with sh-NC or sh-circRNA 100146 no. 1 was examined by MTT assay. OD, optical density. (C) The apoptosis of transfected SW620 and SW480 cells was assessed by flow cytometry assay. (D and E) The migration and invasion of transfected SW620 and SW480 cells were evaluated by transwell assay. (F and G) Western blot assay was conducted to detect the protein levels of PCNA, Bcl-2, E-cad, and MMP9 in transfected SW620 (F) and SW480 (G) cells. *, *P < *0.05.

Cell proliferation, apoptosis, migration, and invasion are closely correlated with tumor progression. To explore the functional effect of circRNA 100146 in CRC progression, SW620 and SW480 cells were transfected with sh-circRNA 100146 no. 1. Methyl thiazolyl tetrazolium (MTT) assay revealed that the cell viability of SW620 and SW480 cells was prominently inhibited by circRNA 100146 silencing (Fig. 2A and B). Flow cytometry assay indicated that silenced circRNA 100146 induced the apoptosis of SW620 and SW480 cells (Fig. 2C). Also, both the migrated and the invaded cell numbers were reduced by circRNA 100146 knockdown in SW620 and SW480 cells (Fig. 2D and E). PCNA (proliferating cell nuclear antigen) is a standard proliferation marker which is commonly used to assess the proliferative ability of cells (20). B-cell lymphoma 2 (Bcl-2), a member of Bcl family, could inhibit the apoptosis (21). E-cadherin (E-cad) and matrix metalloprotein 9 (MMP9) are proteins necessary for tumor invasion and metastasis (22). Hence, we further detected the protein levels to confirm our results. Western blot assay showed that circRNA 100146 silencing led to decreases of PCNA, Bcl-2, and MMP9 levels and a rise of the E-cad level in SW620 and SW480 cells (Fig. 2F and G). All these data suggested that the downregulation of circRNA 100146 impeded cell progression and tumor growth in CRC.

### circRNA 100146 as a molecular sponge antagonized miR-149 availability.

**FIG 3:**
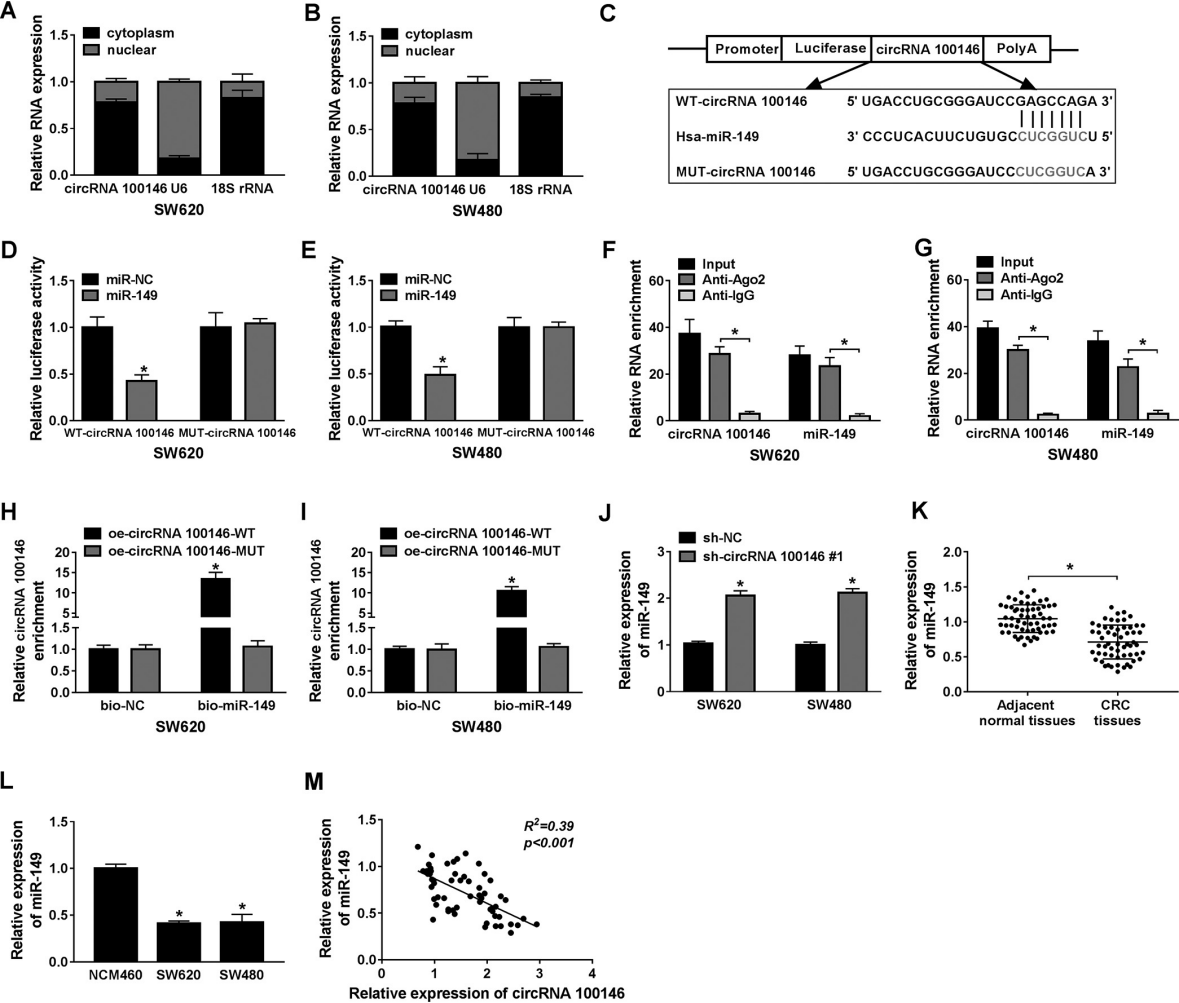
circRNA 100146 bound to miR-149 and negatively regulated miR-149 expression. (A and B) RT-qPCR assay was performed to detect the expression of circRNA 100146, U6 and 18S rRNA in cytoplasm and nuclear. (C) The potential wild-type and mutant binding sites of circRNA 100146 in miR-149 are shown. (D and E) Dual-luciferase reporter assay was conducted in SW620 (D) and SW480 (E) cells cotransfected with miR-NC or miR-149 and WT-circRNA 100146 or MUT-circRNA 100146. (F and G) RIP assay was carried out to measure the levels of circRNA 100146 and miR-149 enrichment in SW620 (F) and SW480 (G) cells. (H and I) The biotinylated miR-147 or miR-NC was, respectively, transfected into SW620 (H) and SW480 (I) cells with WT or MUT circRNA 100146 transcription. The expression levels of circRNA 100146 were tested by qRT-PCR after streptavidin capture. (J) The expression of miR-149 was detected by RT-qPCR assay in SW620 and SW480 cells transfected with sh-NC or sh-circRNA 100146 no. 1. (K) The expression of miR-149 was measured by RT-qPCR assay in CRC tissues and adjacent normal tissues. (L) miR-149 expression in normal colonic epithelial NCM460 and CRC cell lines (SW620 and SW480). (M) The correlation between circRNA 100146 expression and miR-149 expression was determined by Pearson analysis. *, *P < *0.05.

The subcellular localization of circRNA 100146 was confirmed by the nuclear and cytoplasmic mRNA fraction experiment. The results revealed that circRNA 100146 was mainly distributed in the cytoplasm of SW620 and SW480 cells (Fig. 3A and B). circRNA 100146 was predicted by the starBase 3.0 online tool, and it had a potential to bind with miR-149 (Fig. 3C). Then, luciferase vectors (WT-circRNA 100146 and MUT-circRNA 100146) containing binding sequences were constructed, and results from a dual-luciferase reporter assay indicated that overexpression of miR-149 together with WT-circRNA 100146 resulted in an obvious decrease of luciferase activities of SW620 and SW480 cells, while for the MUT-circRNA 100146 group, there was no change between miR-149 and miR-NC (miRNA mimic negative control) (Fig. 3D and E). RNA immunoprecipitation assay (RIPA) demonstrated that not only circRNA 100146 expression but also miR-149 expression was enhanced in the anti-Ago2 complex relative to the anti-IgG complex (Fig. 3F and G). In addition, an RNA pulldown assay was employed and the results showed that a specific pulldown of wild-type circRNA 100146 was observed in the bio-miR-149 group compared with the bio-NC group, while no enrichment was obtained in the mutant circRNA 100146 group (Fig. 3H and I). RT-qPCR assay showed that miR-149 expression was remarkably increased by circRNA 100146 silencing in SW620 and SW480 cells (Fig. 3J). Besides, the level of expression of miR-149 was drastically reduced in CRC tissues and cells (Fig. 3K and L). Pearson analysis showed that there was an inverse correlation between circRNA 100146 expression and miR-149 expression (Fig. 3M). In total, circRNA 100146 absorbed miR-149 to regulate miR-149 expression.

We also evaluated the effects of miR-149 on CRC progression. The cell viability of the CRC cells with miR-149 mimic transfection was reduced in contrast with the viability seen with the cells with miR-NC transfection. Besides, the miR-149 mimic also enhanced the apoptosis rate and the migration and invasion ability of CRC cells. These results demonstrated that miR-149 performs an antitumor function.

### miR-149 inhibition attenuated circRNA 100146 knockdown-mediated antiproliferation, proapoptosis, antimigration, and anti-invasion effects.

**FIG 4:**
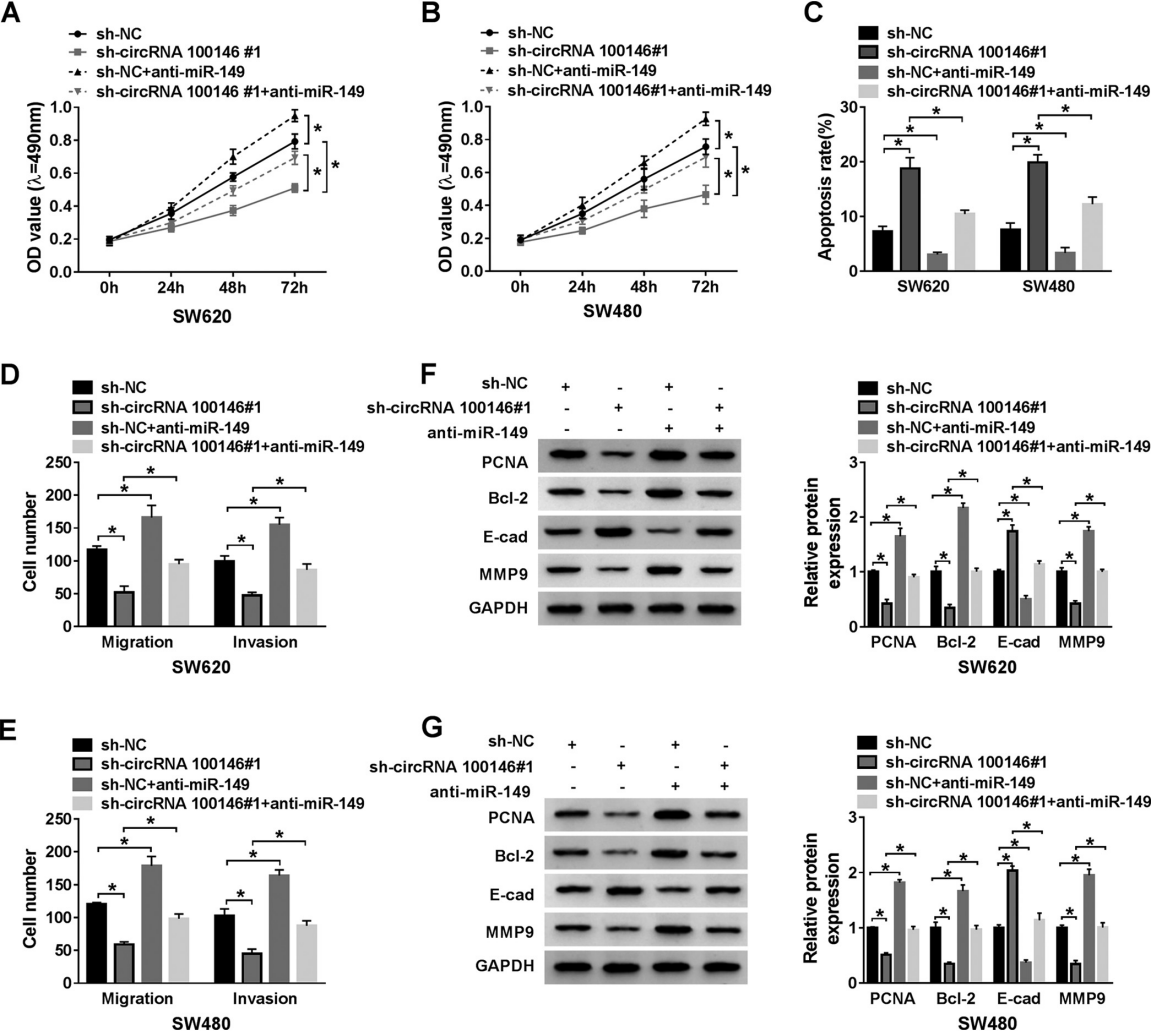
miR-149 reversed the effects of circRNA 100146 on proliferation, apoptosis, migration, and invasion of CRC cells. (A and B) The viability of SW620 (A) and SW480 (B) cells transfected with sh-NC, sh-circRNA 100146 no. 1, sh-NC plus anti-miR-149, or sh-circRNA 100146 no. 1 plus anti-miR-149 was examined by MTT assay. (C) The apoptosis of transfected SW620 and SW480 cells was evaluated by flow cytometry assay. (D and E) The migration and invasion of transfected SW620 (D) and SW480 (E) cells were assessed by transwell assay. (F and G) Western blot assay was conducted to detect the expression of proteins (PCNA, Bcl-2, E-cad, and MMP9) in transfected SW620 (F) and SW480 (G) cells. *, *P < *0.05.

To illuminate the interaction between circRNA 100146 and miR-149 in CRC, SW620 and SW480 cells were transfected with sh-circRNA 100146 no. 1 and/or miR-149. MTT assay indicated that circRNA 100146 silencing repressed the viability of SW620 and SW480 cells and that the effect was abated by introduction of miR-149 inhibition (Fig. 4A and B). Also, the apoptosis of SW620 and SW480 cells was markedly enhanced by circRNA 100146 knockdown, while miR-149 interference reversed the effect (Fig. 4C). Moreover, the number of migrated and invaded cells was reduced by circRNA 100146 knockdown and then was increased following transfection with miR-149 inhibition (Fig. 4D and E). Meanwhile, Western blot assay was conducted to detect the level of proteins related to proliferation, apoptosis, migration, and invasion. The results showed that protein levels of PCNA, Bcl-2, and MMP9 were suppressed by silenced circRNA 100146 and that they were all rescued by cotransfection with miR-149 inhibition. In contrast, the level of E-cad showed a trend opposite that seen with the three proteins named above (Fig. 4F and G). All in all, circRNA 100146 knockdown inhibited proliferation, promoted apoptosis, and blocked migration and invasion via absorbing (“sponging”) miR-149.

### HMGA2 directly interacted with miR-149.

**FIG 5:**
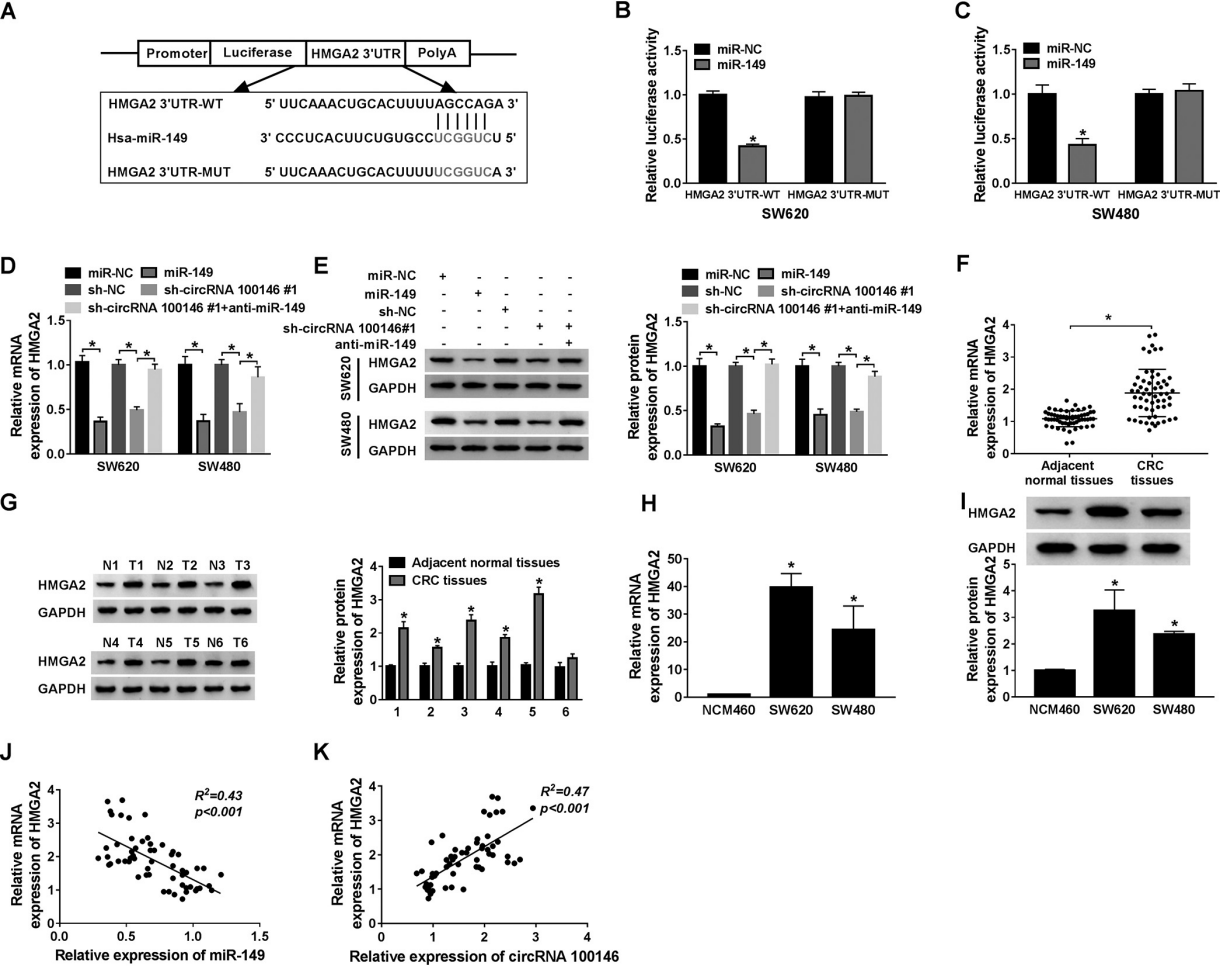
miR-149 targeted to HMGA2 and regulated its expression. (A) The putative sequences with wild-type or mutant binding sites of HMGA2 in miR-149 are exhibited. (B and C) Dual-luciferase reporter assay was performed in SW620 (B) and SW480 (C) cells transfected with miR-NC or miR-149 in HMGA2 3′-UTR-WT and HMGA2 3′-UTR-MUT groups. (D and E) The mRNA (D) and protein (E) levels of HMGA2 were measured in SW620 and SW480 cells transfected with miR-NC, miR-149, sh-NC, sh-circRNA 100146 no. 1, or sh-circRNA 100146 no. 1 plus anti-miR-149 by RT-qPCR and Western blot assays, respectively. (F and G) The mRNA (F) and protein (G) levels of HMGA2 were detected in CRC tissues and adjacent normal tissues by RT-qPCR and Western blot assays, respectively. (H and I) The mRNA (H) and protein (I) levels of HMGA2 were detected in colonic epithelial NCM460 and CRC cell lines (SW620 and SW480) by RT-qPCR and Western blot assays, respectively. (J) The correlation between miR-149 expression and HMGA2 expression was determined by Pearson analysis. (K) The correlation between circRNA 100146 and HMGA2 expression was determined by Pearson analysis. *, *P < *0.05.

starBase 3.0 was used to predict the target of miR-149, and the results showed that HMGA2 had binding sequences with miR-149 (Fig. 5A). To confirm the prediction, HMGA2 3′-UTR-WT and HMGA2 3′-UTR-MUT luciferase vectors were constructed. Dual-luciferase reporter assay results showed that the activities of SW620 and SW480 cells were sharply decreased by transfection with miR-149 compared with the control in the HMGA2 3′-UTR-WT group and showed no change in the HMGA2 3′-UTR-MUT group (Fig. 5B and C). Then, RT-qPCR and Western blot assays indicated that both HMGA2 mRNA and protein expression levels were reduced by miR-149 overexpression. Also, HMGA2 expression was decreased by circRNA 100146 knockdown and that it recovered following transfection with anti-miR-149 (Fig. 5D and E). Further, the mRNA and protein expression levels of HMGA2 were markedly elevated in CRC tissues relative to adjacent normal tissues (Fig. 5F and G). Moreover, HMGA2 mRNA and protein levels were both increased in SW620 and SW480 cells compared with NCM460 cells (Fig. 5H and I). Pearson analysis revealed that there was a negative correlation between miR-149 expression and HMGA2 expression and a positive correlation between circRNA 100146 expression and HMGA2 expression (Fig. 5J and K). Besides, the effect of miR-149 on HMGA2 stability was also assessed. After treatment with 4 μM actinomycin D for 6 h, transfection of miR-149 significantly reduced the stability of HMGA2 while it was increased by anti-miR-149. Overall, miR-149 directly targeted HMGA2 and inversely regulated HMGA2 expression.

### Restoration of HMGA2 expression completely rescued the inhibitory effect of downregulation of circRNA 100146 in CRC cells.

**FIG 6:**
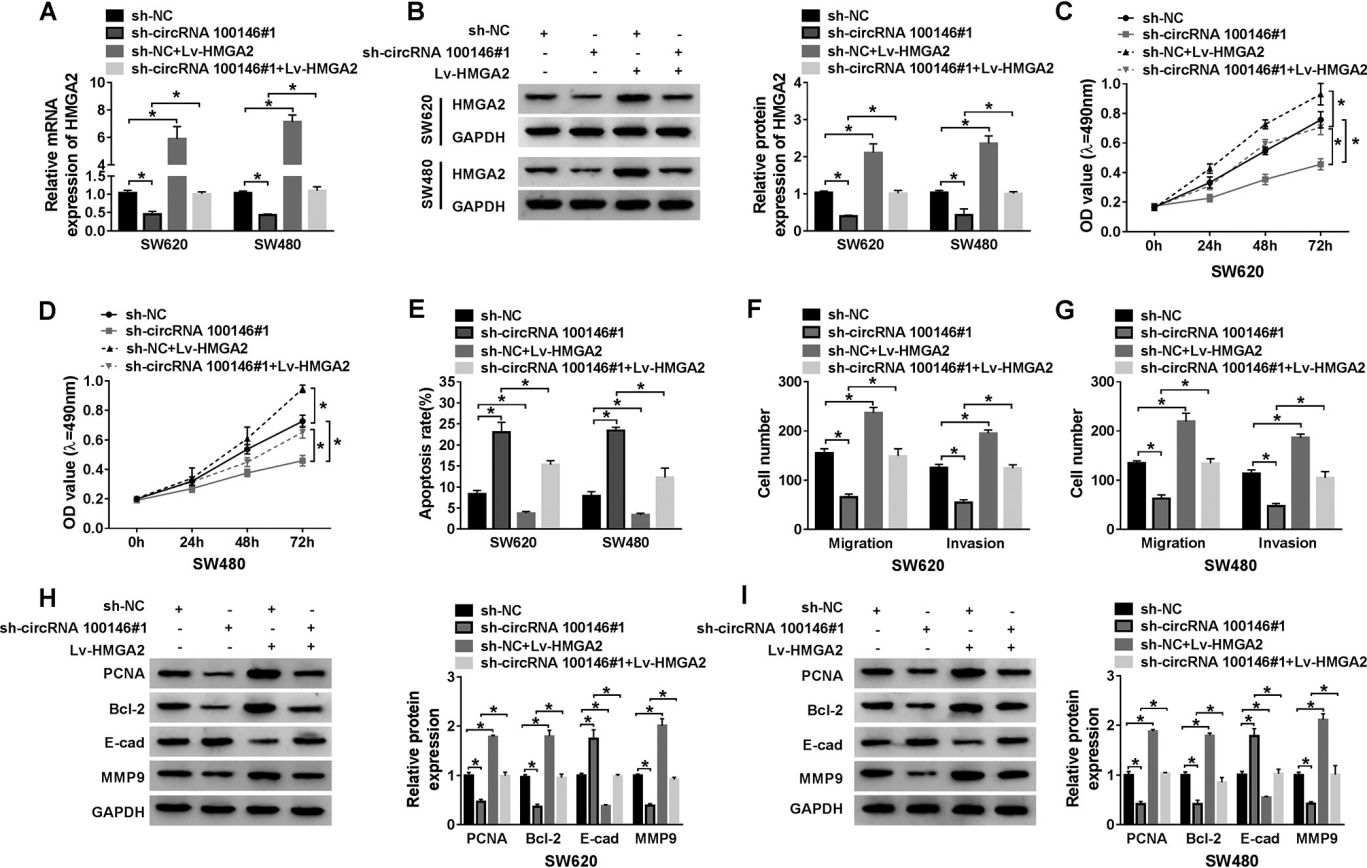
circRNA 100146 knockdown inhibited cell growth by regulating HMGA2 expression. SW620 and SW480 cells were transfected with sh-NC, sh-circRNA 100146 no. 1, sh-NC plus Lv-HMGA2, or sh-circRNA 100146 no. 1 plus Lv-HMGA2. (A and B) The mRNA (A) and protein (B) levels of HMGA2 were detected in SW620 and SW480 cells upon transfection. (C and D) MTT assay was carried out to examine the cell viability of transfected SW620 (C) and SW480 (D) cells. (E) The apoptosis of transfected SW620 and SW480 cells was assessed by flow cytometry assay. (F and G) The migration and invasion of transfected SW620 (F) and SW480 (G) cells were evaluated by transwell assay. (H and I) Proteins levels of PCNA, Bcl-2, E-cad, and MMP9 were examined by Western blot assay in transfected SW620 (H) and SW480 (I) cells. *, *P < *0.05.

To further explore the interaction between circRNA 100146 and HMGA2 in CRC, RT-qPCR and Western blot assays were conducted to detect the expression of HMGA2. The results showed that both mRNA and protein levels of HMGA2 were decreased by circRNA 100146 silencing and were enhanced by HMGA2 upregulation in SW620 and SW480 cells, which were abrogated by cotransfection with 100146 silencing and HMGA2 upregulation (Fig. 6A and B). MTT assay showed that the proliferation of SW620 and SW480 cells was inhibited by silenced circRNA 100146 and was promoted by HMGA2 overexpression and that cell viability was rescued when cotransfection was performed with silenced circRNA 100146 and HMGA2 overexpression (Fig. 6C and D). However, the apoptosis of SW620 and SW480 cells showed the opposite trend with respect to proliferation (Fig. 6E). Transwell assay indicated that the migration and invasion of SW620 and SW480 cells were blocked by circRNA 100146 silencing and were enhanced by HMGA2 overexpression and that both were abated by cotransfection with circRNA 100146 silencing and HMGA2 overexpression (Fig. 6F and G). Western blot assay revealed that protein levels of PCNA, Bcl-2, and MMP9 were all reduced by circRNA 100146 knockdown and were increased by HMGA2 upregulation, while their levels were all restored by cotransfection with circRNA 100146 knockdown and HMGA2 upregulation. In contrast, the trend of E-cad expression was the inverse of that seen with PCNA, Bcl-2, and MMP9 (Fig. 6H and I). On the basis of the results presented above, we speculate that circRNA 100146 silencing inhibits CRC progression through allowing miR-149 binding to 3′-UTR of HMGA2 mRNA.

To confirm this conjecture, an overexpression vector with full-length HMGA2 mRNA was constructed, with the 3′-UTR harboring a mutation in the miR-149 binding sites. SW620 and SW480 cells were transfected with sh-NC, sh-circRNA 100146 no. 1, sh-NC plus Lv-HMGA2-MUT, or sh-circRNA 100146 no. 1 plus Lv-HMGA2-MUT. We first detected the expression level of HMGA2 in the transfected cells. Transfection of sh-circRNA 100146 no. 1 inhibited the mRNA and protein levels of HMGA2 in an obvious manner in contrast to the results seen with the sh-NC group. The mRNA and protein levels of HMGA2 were greatly increased in cells with sh-NC plus Lv-HMGA2-MUT or sh-circRNA 100146 no. 1 plus Lv-HMGA2-MUT transfection. We also detected the effects of Lv-HMGA2-MUT on sh-circRNA 100146 no. 1-mediated cell viability, apoptosis, migration, and invasion. Differently from the cells with sh-circRNA 100146 no. 1 plus Lv-HMGA2 transfection, the cotransfection of sh-circRNA 100146 no. 1 and mutant HMGA2 had little impact on cell viability, apoptosis, migration, and invasion compared with the cell with sh-NC plus Lv-HMGA2-MUT transfection. This suggested that the mutation in HMGA2 3′-UTR that contains the binding sites with miR-149 eventually affected its revision effects on sh-circRNA 100146 no. 1 function, which confirmed our conjecture. In brief, these results indicated that circRNA 100146 silencing allows miR-149 binding to the 3′-UTR of HMGA2 mRNA to inhibit CRC progression.

### sh-circRNA 100146 limited CRC progression by the miR-149/HMGA2 axis *in vivo*.

**FIG 7:**
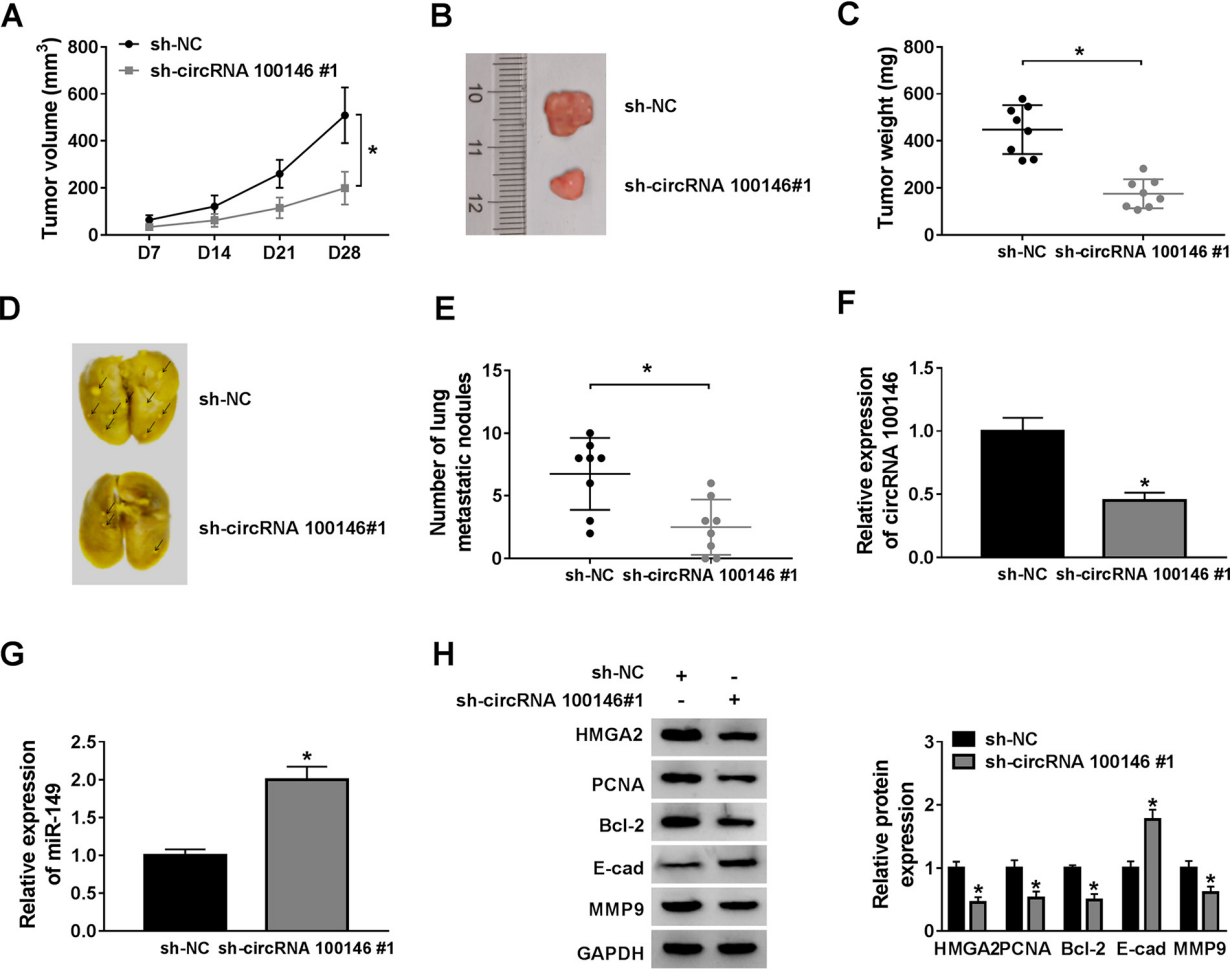
sh-circRNA 100146 limited CRC progression by miR-149/HMGA2 axis *in vivo*. SW620 cells with circRNA 100146 stable knockdown were injected into the nude mice. At 28 days postinoculation, the mice was killed. (A to C) Tumor volumes (A) were measured, and separated tissues were used for photographing (B) and measurement (C). (D and E) For the metastasis experiment, exhibited lung metastatic nodules were pictured (D) and counted (E). (F and G) RT-qPCR assay was used for detecting the expression of circRNA 100146 (F) and miR-149 (G). (H) Western blot assay was carried out for measuring protein expression on HMGA2, PCNA, Bcl-2, E-cad, and MMP9. *, *P < *0.05.

SW620 cells were transfected with sh-circRNA 100146 no. 1 or sh-NC. The SW620 cells with circRNA 100146 stable knockdown were injected into nude mice to establish a xenograft model of CRC in the nude mice. All mice were killed, and tumor samples were separated for measurement of weight after day 28 postinoculation. As shown in Fig. 7A, the mice in the sh-circRNA 100146 no. 1 group possessed a downward-trending tumor volume compared with the mice in the sh-NC group. Besides, we pictured the separated tissues (Fig. 7B), and the statistical results showed that sh-circRNA 100146 no. 1 administration in mice limited tumor weight, compared with sh-NC administration (Fig. 7C). For the experiment investigating metastases, transfected SW620 cells were intravenously injected into the tail of nude mice and changes in tumors (Fig. 7D) and lung metastatic nodules (Fig. 7E) of mice were recorded. As shown in Fig. 7E, few metastatic nodules were seen in mice after intravenous injection with sh-circRNA 100146 no. 1, compared to the sh-NC injection. Furthermore, tissue analyses showed suppression of circRNA 100146 (Fig. 7F) and promotion of miR-149 (Fig. 7G) when mice suffered from sh-circRNA 100146 no. 1 injection, compared to injection with sh-NC. Additionally, injection of sh-circRNA 100146 no. 1 led to restrained expression of HMGA2, PCNA, Bcl-2, and MMP-9 and enhanced expression of E-cad, compared with its own negative controls (Fig. 7H). Taken together, sh-circRNA 100146 limited CRC progression by the miR-149/HMGA2 axis *in vivo*.

## DISCUSSION

The high mortality and poor prognosis of CRC patients were mainly due to the late diagnosis and extended metastasis. Previous studies showed that over 90% of the 5-year survival rate was found in patients in the early stage of CRC, while the survival rate of patients with metastasis was sharply decreased to lower than 10% (23, 24). Mounting evidence has disclosed the crucial roles of circRNAs in development and progression of cancers (25). However, there are limited studies on their roles in CRC.

In the current study, the expression level of circRNA 100146 in CRC was revealed. Expression of circRNA 100146 was enhanced in CRC tissues and cells in contrast with the adjacent normal tissues and normal cells. Surprisingly, the level of expression of circRNA 100146 in positive-metastasis tissues and advanced tumor stages (III and IV) was much higher than in negative metastasis tissues and early tumor stages (I and II). This indicated that the level of expression of circRNA 100146 might nearly correlate with the progression of CRC.

circRNA 100146 derived from exon 5 and exon 6 of the EIF3I gene. Research has uncovered that it was highly expressed in non-small-cell lung cancer (NSCLC) cells and that circRNA 100146 could interact with miR-361-3p and miR-615-5p as a competing endogenous RNA (ceRNA) to modulate cell progression of NSCLC (9). Since the EIF3I gene acts as an oncogene and has been shown to be involved in the progression of multiple cancers, our priority is to exclude the possibility of a function of EIF3I in CRC. CRC cells were transfected with sh-NC, sh-circRNA 100146 no. 1, or sh-circRNA 100146 no. 2, and the levels of expression of circRNA 100146 and its host EIF3I gene were determined. Results showed that transfection of sh-circRNA 100146 no. 1 or sh-circRNA 100146 no. 2 decreased the expression of circRNA 100146 to various degrees but had no effect on EIF3I expression. They further indicated that the small interfering RNA (siRNA) that we used affected only the expression of circRNA 100146. Thus, the changes in cell biological processes that were induced by circRNA 100146 silencing were irrelevant to EIF3I. Subsequently, the effects of circRNA 100146 on CRC were detected. Our results showed that circRNA 100146 knockdown led to inhibition of proliferation, migration, and invasion and promotion of apoptosis of CRC cells. These data suggested that circRNA 100146 was involved in the tumorigenesis and development of CRC.

circRNAs could function as competing endogenous RNAs (ceRNAs) to bind with miRNA to exert various biological functions (26). In order to search for the target of circRNA 100146, we employed the starBase 3.0 online tool to predict its potential target. Interestingly, miR-149 had the binding sequences corresponding to circRNA 100146. The results from dual-luciferase reporter, RIP, and biotinylated RNA pulldown assays supported this prediction. Notably, the level of expression of miR-149 was decreased in CRC tissues and cells, which was consistent with the previous study (27). And miR-149 expression was negatively correlated with circRNA 100146 expression. miR-149 exerted a tumor suppressor effect as its overexpression inhibited cell viability, migration, and invasion but promoted cell apoptosis. In addition, miR-149 inhibition could reverse the effects of circRNA 100146 knockdown on the proliferation, apoptosis, migration, and invasion of CRC cells. All of the data described above implied that circRNA 100146 absorbed miR-149 to regulate cell progression in CRC.

HMGA2 is an important transcription factor in CRC (28, 29), and it was identified as a target gene of miR-149 based on the predicted results provided by the starBase 3.0 website tool. And the results were supported by the dual-luciferase reporter and RIP assay. Results reported previously by Mansoori et al. indicated that HMGA2 was highly expressed and promoted cell proliferation and migration in CRC cells (30). In accordance with these results, we measured the level of expression of HMGA2 and found that HMGA2 expression was prominently increased in CRC patients and cells. Also, HMGA2 expression was inversely correlated with miR-149 expression and was positively correlated with circRNA 100146 expression in CRC. More importantly, functional analysis indicated that HMGA2 overexpression rescued the effects caused by circRNA 100146 knockdown in proliferation, apoptosis, migration, and invasion of CRC cells. The mutations affecting the miR-149 binding sites of the HMGA2 3′-UTR caused it to lose the ability to reverse the inhibition effects induced by circRNA 100146 silencing. Taking the data together, circRNA 100146 regulated cell progression of CRC cells by targeting miR-149 to modulate HMGA2 expression.

Besides, circRNA 100146 silencing suppressed tumor growth and pulmonary metastasis, indicating the possibility of using circRNA 100146 as a therapeutic target for CRC. Although several important findings on the effect and underlying mechanism of circRNA 100146 in CRC have been explored in this research, the molecular mechanisms of circRNA 100146 regulation in CRC still need further in-depth understanding. Is the regulatory function of circRNA 100146 also observable in clinical practice? What are the pathways downstream of circRNA 100146/miR-149/HMGA2 regulation in CRC? To work around these issues, future work will be required to further elucidate pathways downstream of circRNA 100146/miR-149/HMGA2 regulation in CRC and the connection between circRNA 100146 expression and clinical advances.

### Conclusion.

circRNA 100146 was highly expressed in CRC patients and cells. circRNA 100146 silencing hindered cell progression in CRC *in vitro* and *in vivo.* Moreover, miR-149 was a target of circRNA 100146 and silenced circRNA146-repressed cell progression by sponging miR-149. Also, miR-149 directly bound to HMGA2 and reduced HMGA2 expression. miR-149 inhibition relieved circRNA 100146 knockdown-mediated inhibitory effects on HMGA2 expression. Furthermore, circRNA 100146 knockdown restrained the progression of CRC cells via targeting miR-149 to downregulate HMGA2 expression. To some extent, these findings revealed the role and potential mechanism of circRNA 100146 and provided evidence that circRNA 100146 served as an early diagnostic biomarker for detection of CRC.

## MATERIALS AND METHODS

### Tissues and cell culture.

All patient tissues were collected from patients in the Department of General Surgery, Second Ward, Rizhao People's Hospital, and written informed consent was signed prior to this study. Before collection of the samples, patients did not receive any treatment. All tissues (*n* = 58) were classified according to the metastasis and stage of disease of patients and then stored in liquid nitrogen immediately. Table 1 presents the clinicopathological characteristics of the patients. All experiments were permitted by the Ethics Committee of Department of General Surgery, Second Ward, Rizhao People's Hospital.

**TABLE 1:** The clinicopathological features in 58 patients with CRC

Parameter	No.
Gender	
Male	26
Female	32
Age (yrs)	
<60	28
≥60	30
Histological grade	
Healthy/moderately healthy	43
Poor health	15
TNM stage	
I + II	25
III	22
IV	11
Lymph node metastasis	
Positive	27
Negative	31

SW620 and HCT116 cells were obtained from American Type Culture Collection (ATCC; Manassas, VA). The SW480 cell line was purchased from the Cell Bank of the Chinese Academy of Sciences (Shanghai, China). The LOVO cell line was provided by Procell Life Science and Technology (Wuhan, China). The normal colonic epithelial NCM460 cell line, which has been proven as an antitumor control model, was provided by the Cell Bank of Type Culture Collection (Shanghai, China). All cell lines were incubated in RPMI 1640 medium (HyClone, South Logan, UT)–10% fetal bovine serum (FBS; Gibco, Rockville, MD) and were cultured in an incubator at 37°C in a 5% CO_2_ atmosphere.

### Cell transfection.

To obtain small hairpin RNAs (shRNAs) uniquely targeting only the circRNA and not the linear RNA, the predicted sense strand and antisense strand were linked by a loop (9 nt or 10 nt) to form stable hairpin RNAs known as sh-circRNA 100146 no. 1 and sh-circRNA 100146 no. 2. And the transfection efficiency was determined by RT-qPCR. circRNA 100146 silencings (sh-circRNA 100146 no. 1 and sh-circRNA 100146 no. 2) and the control silencing (sh-NC), miR-149 overexpression (miR-149) and its control (miR-NC), antisense RNA against miR-149 (anti-miR-149), lentiviral vector of HMGA2 (Lv-HMGA2), and lentiviral vector of HMGA2 with 3′-UTR mutant (Lv-HMGA2-MUT) were obtained from GenePharma (Shanghai, China). All vectors or oligonucleotides were transfected into colorectal cancer cells by the use of Lipofectamine 2000 (Invitrogen, Carlsbad, CA). Then, the transfected cells were cultured in an incubator at 37°C.

### RNase R digestion and gene stability detection.

In brief, 5 μg of the extracted RNA was digested by the use of 20 U of RNase R (Geneseed, Guangzhou, China) in the reaction buffer at 37°C for 30 min. Another digestion assay was performed for the control with digestion buffer only, followed by RT-qPCR for circRNA and mRNA to determine the levels of expression of circRNA and mRNA.

For detection of gene stability, the transfected cells were treated with 4 μM actinomycin D for 0 h, 2 h, 4 h, and 6 h, and total RNA was collected, followed by RT-qPCR assay.

### Quantitative real-time PCR (RT-qPCR).

Tissues from colorectal cancer patients were ground under liquid nitrogen, and cells were collected after 48 h of cultivation. All tissues and cells were added to TRIzol reagent (Invitrogen, Carlsbad, CA) for total RNA isolation. The quality and concentration of RNA were determined by the use of a NanoDrop 2000 spectrophotometer (Thermo Fisher Scientific, Rockford, IL). Then, an All-in-One miRNA first-strand cDNA synthesis kit (GeneCopoeia, Rockville, MD) was used to synthesize cDNA for miRNA analysis, while for circRNA and mRNA analysis, TransScript First-Strand cDNA Synthesis Supermix (TransGen Biotech, Beijing, China) was selected to synthesize cDNA. RT-qPCR was carried out with an All-in-One miRNA RT-qPCR detection kit (GeneCopoeia). The results of RT-qPCR assay were standardized with the threshold cycle formula 2^−ΔΔ^*^CT^*. Primers for circRNA 100146, miR-149, HMGA2, glyceraldehyde-3-phosphate dehydrogenase (GAPDH), and U6 were synthesized by Sangon Biotech (Shanghai, China), and the sequences were as follows: for circRNA 100146, 5′-GAGCTCAACCAGTATAGTGCC-3′ (forward) and 5′-ACATGATGATGTTGCCCCCAA-3′ (reverse); for miR-149, 5′-GCAGGGGAGTGAAGACAC-3′ (forward) and 5′-GGTCCAGTTTTTTTTTTTTTTTCTC-3′ (reverse); for HMGA2, 5′-CTCAAAAGAAAGCAGAAGCCACTG-3′ (forward) and 5′-TGAGCAGGCTTCTTCTGAACAACT-3′ (reverse); for GAPDH, 5′-TCACCACCATGGAGAAGGC-3′ (forward) and 5′-GCTAAGCAGTTGGTGGTGCA-3′ (reverse); for U6, 5′-ATTTGATGGGTGAGGAATGGGTT-3′ (forward) and 5′-CGCTTCACGAATTTGCGTGTCAT-3′ (reverse). To confirm the specificity of the primers, a qPCR assay was performed and the primer with the single correct size of electrophoretic strip was chosen.

### MTT assay.

The transfected colorectal cancer cells were seeded into 96-well plates and were cultivated in an incubator at 37°C for 24 h, 48 h, or 72 h. Then, all cells were added to 0.5 mg/ml methyl thiazolyl tetrazolium (MTT) solution (Sigma, St. Louis, MO) for another 4 h of culture. Next, the formazan was collected and solubilized with dimethyl sulfoxide (DMSO; Sigma). Finally, a microplate reader (Bio-Rad, Hercules, CA) was used to measure the absorbance at 490 nm.

### Flow cytometry assay.

An annexin V-fluorescein isothiocyanate (FITC)–propidium iodide (PI) apoptosis detection kit (Solarbio, Beijing, China) was used to detect cell apoptosis. After cultivation for 48 h, colorectal cancer cells were harvested and suspended with binding buffer. Then, the cell suspension was incubated with annexin V-FITC and PI solution for 10 min under conditions of a dark environment. A flow cytometer (BD Biosciences, San Jose, CA) was employed to analyze the apoptotic cells.

### Transwell assay.

Both cell migration and invasion were detected by transwell assay. The chambers could be directly used for the analysis of cell migration, while, for cell invasion analysis, transwell chambers were covered with Matrigel (Becton, Dickinson, Franklin Lakes, NJ) in advance. Transfected colorectal cancer cells were placed into the upper chambers. Then, crystal violet was added into the upper chambers for 5 min of incubation. Cells located at the upper chambers were wiped with a cotton bud, and cells through the membranes were counted by microscope (Olympus, Tokyo, Japan) with more than three random views.

### Western blot assay.

After cultivation for 48 h, transfected colorectal cancer cells were collected and lysed with RIPA lysis buffer (Beyotime, Shanghai, China). Then, the quality and quantity of total proteins were measured with a bicinchoninic acid (BCA) protein assay kit (Cowbiotech, Beijing, China). Subsequently, total protein samples were separated with sodium dodecyl sulfate-polyacrylamide gel electrophoresis (SDS-PAGE) gels and then blotted onto polyvinylidene difluoride (PVDF) membranes (Millipore, Bedford, MA). Next, the blocking experiment was conducted by adding 5% bovine serum albumin (BSA) into the membranes, and then the membranes were incubated overnight at 4°C with primary antibodies against proliferating cell nuclear antigen (PCNA) (ab29; Abcam, Cambridge, MA) (1 μg/ml), B-cell lymphoma 2 (Bcl-2) (ab32124; Abcam) (1/1,000), E-cadherin (E-cad) (ab1416; Abcam) (1/50), matrix metalloprotein 9 (MMP9) (ab38898; Abcam) (1/1,000), HMGA2 (ab97276; Abcam) (1/1,000), or GAPDH (ab37168; Abcam) followed by incubation with a horseradish peroxidase (HRP)-conjugated second antibody (ab6721; Abcam), and the bands were scanned using Image Lab software (Bio-Rad).

### Murine xenograft assay.

The nude mice were all obtained from Vital River Laboratory Animal Technology (Beijing, China). All the animal experiments were permitted by the Animal Research Committee of Department of General Surgery, Second Ward, Rizhao People's Hospital, and conducted following the guidelines of the National Animal Care and Ethics Institution. SW620 cells were transfected with sh-circRNA 100146 no. 1 or with sh-NC. Then, the nude mice were infected subcutaneously with stably transfected cells. After cultivation for 7 days, 14 days, 21 days, and 28 days, tumor width and length were measured. The following formula was used to calculate tumor volume: width^2^ × length/2. At day 28 postinoculation, all mice were killed, and tumor samples were taken for measurement of weight. For metastasis experiments, transfected cells at a density of 2 × 10^6^ were intravenously injected in the tail of the nude mice (*n* = 8). The lung metastases were counted after 8 weeks. The changes in the primary tumor and lung metastatic tumor in mice were recorded.

### Dual-Luciferase reporter assay.

The potential binding sites of miR-149 and circRNA 100146 or HMGA2 were predicted by the use of the starBase 3.0 online tool. The circRNA 100146 and HMGA2 wild-type sequences were amplified and inserted into luciferase pGL3 vector (Promega, Madison, WI). The mutant sequences of circRNA 100146 and HMGA2 were designed, synthesized, and cloned into pGL3 vector. All vectors were cotransfected into colorectal cancer cells with miR-NC or miR-149 using Lipofectamine 2000 transfection reagent. At 48 h posttransfection, the luciferase activities of transfected SW620 and SW480 cells were detected by the use of a luciferase reporter assay kit (Promega).

### RNA immunoprecipitation (RIP) assay.

miRNA was encapsulated mainly by Ago2 protein when it formed the binding of the RNA-induced silencing complex (RISC). Thus, when the Ago2 protein was integrated with anti-Ago2 antibody, the bonding miRNA as well as the RISC-bound circRNA and mRNA would also be pulled down. IgG antibody was used as a negative control to rule out false-positive results. Therefore, we chose the Ago2 RIP assay to detect the target relationship (31). A Magna RNA immunoprecipitation kit (Millipore, Billerica, MA) was used to conduct the RIP assay. SW620 and SW480 cells were transfected with miR-149 or circRNA 100146. Then, cells were harvested and lysed in RIP buffer containing magnetic beads conjugated with Ago2 (ab32381; Abcam) (5 μg/ml) or IgG (ab6721; Abcam) (1/1,000) antibody. Next, RNAs which bound to Ago2 and IgG were isolated according to the RNA extraction protocol and the levels of enrichment of circRNA 100146 and miR-149 were analyzed by RT-qPCR assay.

### Biotinylated RNA pulldown assay.

The biotinylated RNA pulldown assay was conducted to determine the interaction between circRNA 100146 and miR-149. In brief, for miR-149 pulldown with circRNA 100146, a biotinylated-miR-149 (bio-miR-149) probe was synthesized by GenePharma (Shanghai, China). C-1 magnetic beads (Life Technologies) were employed to conduct the RNA pulldown assay. The C-1 magnetic beads were incubated with bio-miR-149, followed by incubation with bio-miR-149 or bio-miR-NC at 4°C overnight. After washing with the wash buffer was performed, the RNA complexes were eluted for real-time qPCR analysis.

### Statistical analysis.

The statistical analysis was carried out using GraphPad Prism 7 (GraphPad Inc., La Jolla, CA). The data are presented as means ± standard deviations (SD) of results from at least three independent experiments. Comparisons of two groups were performed by Student's *t* test, and multiple comparisons were performed by one-way analysis of variance (ANOVA). A *P* value of *<*0.05 was regarded as representing a statistically significant difference.
